# Clinical characterization and etiological insights: a small cohort study of pulmonary infections caused by *Microascus* spp. in critically ill patients

**DOI:** 10.3389/fmicb.2025.1632354

**Published:** 2025-10-02

**Authors:** Ting Zhang, Jian-Jun Cheng, Ru-Ru Bi, Jun-Mei Zhu, Li-Ting Zhou, Yan Chen, Qing-Zhen Han

**Affiliations:** Center of Clinical Laboratory Medicine, The Fourth Affiliated Hospital of Soochow University (Suzhou Dushu Lake Hospital), Suzhou, Jiangsu, China

**Keywords:** *Microascus* spp., hematopoietic stem cell transplantation, antifungal susceptibility testing, MALDI-TOF, multilocus sequence typing, environmental monitoring

## Abstract

*Microascus* spp., globally distributed fungi, are increasingly recognized as causative agents of rare and refractory invasive infections in immunocompromised populations, with approximately 50 cases reported worldwide. This study aimed to characterize the epidemiological features, antifungal resistance profiles, and identification strategies for *Microascus*-associated pulmonary infections. Ten *Microascus* isolates were collected from respiratory specimens of patients with pulmonary infections (2021–2024). Clinical characteristics were analyzed, and antifungal susceptibility testing (AFST) was performed following CLSI M38-A3 guidelines. Taxonomic identification integrated MALDI-TOF mass spectrometry, and multilocus phylogenetic analysis (*ITS/EF-1α/TUB*). Nine of ten cases occurred in hematopoietic stem cell transplant (HSCT) recipients, with concurrent infections by cytomegalovirus (7/9), *Pseudomonas aeruginosa* (5/9), *Corynebacterium striatum* (5/9), or *Aspergillus* spp. (5/9). Three patients succumbed to refractory infections. Morphologically, colonies exhibited olive-to-black concentric rings, floccose hyphae with dark granules, and basally swollen conidiophores producing oval or pear-shaped conidia in chains. Nine isolates were *M. gracilis*, and one was *M. cirrosus.* All strains demonstrated resistance to fluconazole, amphotericin B, and flucytosine (MIC >64 μg/mL) but high sensitivity to terbinafine (MIC ≤0.125 μg/mL). MALDI-TOF accurately identified *M. gracilis* (100%), while *M. cirrosus* required sequencing for confirmation. Multilocus sequence typing revealed a monophyletic cluster among *M. gracilis* isolates. *Microascus* spp. represent underdiagnosed pathogens in HSCT-associated fungal pneumonia, often complicated by polymicrobial infections. Terbinafine demonstrates promising *in vitro* efficacy against multidrug-resistant strains. A multimodal diagnostic approach combining morphology, MALDI-TOF, and sequencing is essential for species-level identification.

## Introduction

*Microascus* spp., classified within the family Microascaceae (order Microascales, class Sordariomycetes), represent emerging opportunistic pathogens associated with life-threatening infections globally. These fungi predominantly afflict immunocompromised hosts, including hematopoietic stem cell transplant (HSCT) recipients, critically ill patients, and individuals undergoing prolonged immunosuppressive therapy (Schoeppler et al., 2015). High mortality rates persist due to diagnostic delays, intrinsic antifungal resistance, and limited therapeutic options. Clinical manifestations range from localized cutaneous abscesses to disseminated infections involving lungs, sinuses, cardiac valves, and the central nervous system, often demonstrating poor prognosis despite aggressive interventions (Sandoval-Denis et al., 2016). Recent years have witnessed increased reports of severe pulmonary, hematogenous, and deep-tissue infections, paralleling the growing population of transplant recipients (Baddley et al., 2000; Miossec et al., 2011; Ding et al., 2020; Brasch et al., 2018). However, systematic analyses of clinical features and microbiological characteristics remain scarce, with current literature predominantly limited to case reports.

Ecologically diverse, *Microascus* spp. typically exhibit saprophytic or weakly parasitic lifestyles, colonizing air, soil, animal feces, plant debris, and humid environments. M. Sandoval-Denis et al. conducted a study on 141 clinical and environmental isolates by integrating morphological, physiological, and multigene sequence analyses of ITS, LSU, EF-1α, and β-tubulin genes. Based on these analyses and applying the Genealogical Concordance Phylogenetic Species Recognition (GCPSR) criterion, they revealed that *Microascus* and *Scopulariopsis* belong to two distinct evolutionary lineages. Additionally, they analyzed 54 species from 12 genera within the family *Microascaceae*, as well as one species from the family *Graphiaceae*. The results demonstrated that *Microascus* and *Scopulariopsis* are polyphyletic genera, with their species distributed across multiple distantly related clades (Sandoval-Denis et al., 2016). According to MycoBank (as of September 2025), 98 species names are currently accepted in *Microascus*; however, some of these taxa are based on lost specimens or invalid names. To better delineate species boundaries and elucidate evolutionary relationships within the genus, Wei et al. (2024) conducted multi-locus phylogenetic analyses using a combined dataset of four gene regions (ITS, LSU, *tef1*, and *tub2*), incorporating expanded taxon sampling and morphological assessments. Their results resolved 49 phylogenetic species in *Microascus* with high statistical support. Differentiated by ascus morphology (peridium structure) and ascospore characteristics (size, symmetry, surface ornamentation) [drfungus.org/*Microascus* Species]. Critical gaps persist in morphological identification criteria, antifungal susceptibility patterns, and genomic characterization, contributing to diagnostic and therapeutic challenges.

Our team recently reported China’s first fatal *M. cirrosus*-associated invasive pulmonary infection in an HSCT recipient, with whole-genome sequencing data deposited in NCBI (PRJNA835605) (Cheng et al., 2023). Subsequent clinical surveillance identified nine additional *M. gracilis* infections. This study systematically analyzes the clinical and microbiological profiles of *Microascus*-related severe pneumonia to inform improved management strategies.

## Materials and methods

### Strain collection and clinical data

Ten *Microascus* isolates (preliminarily identified by Bruker MALDI-TOF MS and confirmed via Sanger sequencing) were collected from patients at the Fourth Affiliated Hospital of Soochow University (September 2021–September 2024). Clinical data included: (1) Demographics (sex, age, Underlying disease); (2) Time to infection post-transplantation; (3) Antifungal prophylaxis post-transplantation; (4) Specimen type; (5) Imaging findings; (6) Co-pathogens; (7) Antimicrobial treatment strategy; and (8) outcomes (improvement or deterioration).

Case data collection from the literature: Case reports published between 1990 and 2025 were retrieved from the PubMed database using the keywords “*Microascus,*” “infection,” and “human.” Information including patient age, sex, pathogen species, infection site, underlying condition/transplant type, treatment regimen, and clinical outcome was extracted from each eligible report.

### Instruments and reagents

Bruker MALDI-TOF MS (Bruker Daltonics, Germany), SLAN-96P real-time PCR system (Hongshi Medical, China). Reagents: Sabouraud dextrose agar (Autobio, China), lactophenol cotton blue stain (Besio Biotechnology), antifungal agents (amphotericin B, caspofungin acetate, terbinafine, fluconazole, flucytosine; BBI Solutions), and fungal DNA extraction kit (Sangon Biotech).

### Strain isolation and culture

Sputum/bronchoalveolar lavage fluid (BALF) specimens were inoculated onto Sabouraud dextrose agar (SDA) and incubated at 35 °C or 28 °C for 3–5 days. Brown colonies (<1 cm diameter) were subcultured on fresh SDA for morphological characterization. Extended incubation (≤14 days) enabled assessment of colony morphology and microscopic structures. The morphological characteristics were observed under an Olympus CX-33 optical microscope (400× and 1,000× magnification) using lactophenol cotton blue staining, and images were captured with the MShot Image Analysis System software.

### MALDI-TOF identification

According to the filamentous fungi identification SOP recommended by Bruker Daltonics, fungal colonies were liquid-cultured for 48 h, harvested by centrifugation (10,000 ×*g*, 10 min), and washed twice with 1 mL deionized water (vortexed, then centrifuged at 10,000 ×*g* for 10 min). The pellet was resuspended in 300 μL deionized water and 900 μL absolute ethanol, vortexed, and centrifuged (10,000 ×*g*, 10 min). After air-drying, the pellet was treated with 50 μL 70% formic acid (thoroughly mixed), followed by addition of 50 μL acetonitrile and vigorous mixing. The final suspension was centrifuged (10,000 ×*g*, 10 min), and 1 μL supernatant was spotted onto a MALDI target plate, air-dried at room temperature, and overlaid with 1 μL matrix solution (α-cyano-4-hydroxycinnamic acid in 50% acetonitrile/2.5% trifluoroacetic acid). MALDI-TOF MS analysis was performed using the Bruker Biotyper system (Bruker Daltonics) according to the manufacturer’s protocol. Protein profiles were acquired using flexControl 3.4 (Bruker) and analyzed against the MBT Filamentous Fungi Library v2.0.

### DNA extraction and multilocus sequencing

Genomic DNA was extracted using Ezup Column Fungal DNA Kit (Sangon Biotech) from mechanically disrupted hyphae. Three loci—ITS, EF-1α, and β-tubulin—were amplified (50 μL reaction: 95 °C/5 min; 40 cycles of 95 °C/5 s, 52–58 °C/30 s, 72 °C/30 s). Sanger sequencing (Sangon Biotech) results were aligned with GenBank references via Clustal W in MEGA-12. Phylogenetic trees were reconstructed using maximum likelihood (ML) method.

### Antifungal susceptibility testing

Broth microdilution assays were performed per CLSI M38-A2 guidelines. Tested agents included caspofungin acetate (0.125–16 μg/mL), terbinafine (0.125–16 μg/mL), amphotericin B (0.5–64 μg/mL), fluconazole (0.5–64 μg/mL), and flucytosine (0.5–64 μg/mL).

### Environmental monitoring and surface sampling

Environmental and surface samplings were performed according to the WS/T 592-2018 standard (Standard Terminology for Healthcare-associated Infection Management). Air sampling was conducted in the bronchoscopy procedure room using the sedimentation method at five sampling points and one negative control. The exposure time was 15 min per plate. Surface sampling was performed in the bronchoscopy procedure room on three operating table surfaces, and in the respiratory ward on twelve hospital bed surfaces, including bed rails and mattresses. All samples were incubated at 37 °C for 7 days with daily examinations for filamentous fungal growth. Fungal isolates were identified by Matrix-Assisted Laser Desorption/Ionization Time-of-Flight Mass Spectrometry (MALDI-TOF MS) (Bruker Daltonics) following standard protocols.

## Results

### *Microascus* spp. as emerging pathogens in HSCT recipients

Among 49 filamentous fungal isolates from HSCT patients (January 2021–December 2024), *Microascus* spp. accounted for 18.4% (9/49), ranking after *Aspergillus* (61.2%, 30/49) and preceding *Penicillium* (34.7%, 17/49). All nine *Microascus*-positive cases (90%) were HSCT recipients in stable remission (median post-transplant interval: 31 months), while one case involved a COPD exacerbation patient (Table 1).

**Table 1:** Distribution and comparative analysis of filamentous fungi isolated from HSCT patients.

Fungal species^a^	No. of isolates *n* = 49, (%)	Statistical analysis (vs. *Microascus*)	*p-value*
*Aspergillus* spp.	30 (61.2)	Chi-square test	<0.001
*Penicillium* spp.	17 (34.7)	Chi-square test	0.077
*Microascus* spp.	9 (18.4)	/	/
*Cladosporium* spp.	8 (16.3)	Chi-square test	0.791
*Mucorales*	3 (6.1)	Fisher’s exact test	0.163
*Scedosporium* spp.	2 (4.1)	Fisher’s exact test	0.104

a Some patients exhibited mixed fungal infections (*Aspergillus* + *Microascus* or *Aspergillus* + *Penicillium*).

### Clinical profiles and therapeutic outcomes

Ten patients (MG1-MG10; male:female = 4:6; median age = 39 years) exhibited bronchiolitis obliterans on High-Resolution Computed Tomography (HRCT, 100%), with characteristic radiologic findings including bronchial wall thickening (8/10), patchy infiltrates (6/10), and cavitation (2/10). Co-infections were predominantly caused by viral pathogens (CMV, Rhinovirus, 2019-nCoV, Adenovirus, Influenza A) and *Aspergillus* spp. (5/10), as shown in Table 2. Despite receiving multimodal therapy (broad-spectrum antibiotics + antifungals ± antivirals), three patients developed clinical deterioration.

**Table 2:** Clinical characteristics of 10 patients with *Microascus*-positive cultures.

ID (MG)	Sex	Age	Underlying disease	Time to infection	Antifungal prophylaxis*	Specimen	Imaging findings	Co-pathogens	Treatment	Outcome
1	F	22	ALL	50 m	PCZ	BAL	Bilateral pulmonary infiltrates (CT); white necrotic mucosa (bronchoscopy)	CMV, COVID-19, *Pneumocystis*, *Candidal*, *Pseudomonas aeruginosa*	SCF, PCZ, GCV,	Improved
2	F	33	AML	30 m	PCZ	Sputum	Bilateral infiltrates (portable DR)	COVID-19, Adenovirus	CZA, LEV, PCZ, ACV,	Deteriorated
3	F	58	ALL	31 m	PCZ	Sputum	Bilateral patchy opacities & bronchiolitis (CT)	CMV	SCF, MCFG, FCZ,	Improved
4	F	36	AML	23 m	PCZ	Sputum	Bronchiectasis with peribronchial inflammation (CT); viscous secretions (bronchoscopy)	CMV, COVID-19, *E. hormaeche i*, *K. pneumoniae*, *Aspergillus*, *Mucor*, *Candida*	SCF, MEM, TGC, AMB	Improved
5	M	51	CML	30 m	VCZ	Sputum	Bilateral infiltrates; bronchial obstruction by necrotic material (CT)	CMV, COVID-19, Rhinovirus, *Aspergillus*	MEM, TGC, VCZ, GCV	Improved
6	M	85	AECOPD	/	None	Sputum	Bronchial wall thickening; right lobe consolidation (portable DR)	*P. aeruginosa*, *C. striatum*, *Aspergillus*, Influenza A	TZP, LEV, OSV, CSFG	Deteriorated
7	F	39	AML	70 m	VCZ	Sputum	Multifocal consolidations & bronchiectasis (CT)	*K. pneumoniae*, CMV, Rhinovirus, *M. pneumoniae*	SCF, CAZ, BIPM, VCZ, LEV	Improved
8	M	40	MPAL	35 m	CSFG, PCZ	BAL	Right middle & lower lobe infiltrates (CT); frothy sputum (bronchoscopy)	*Nocardia*, CMV, *Aspergillus*	LZD, MEM, CSFG, PCZ, ACV	Deteriorated
9	F	34	AML	24 m	PCZ	BAL	Bronchiectasis with infection (CT)	CMV, *Aspergillus*, *Candida*, *E. coli*	BIPM, LEV, VCZ, ACV	Improved
10	M	57	AML	31 m	ISA	Sputum	Multifocal pneumonic infiltrates (CT)	*E. hormaechei*, *A. hydrophila*, *P. aeruginosa*	TZP, MXF, ISA, GCV	Improved

m, months post-transplantation to *Microascus* detection. *Antifungal prophylaxis: Agents administered prior to Microascus diagnosis. ALL, Acute lymphoblastic leukemia; AML, Acute myeloid leukemia; CML, Chronic myeloid leukemia; MPAL, Mixed-phenotype acute leukemia. Antimicrobial abbreviations: SCF, Cefoperazone-sulbactam; MEM, Meropenem; TGC, Tigecycline; AMB, Amphotericin B; TZP, Piperacillin-tazobactam; MXF, Moxifloxacin; ISA, Isavuconazole; GCV, Ganciclovir; VCZ, Voriconazole; ACV, Acyclovir; BIPM, Biapenem; LEV, Levofloxacin; PCZ, Posaconazole; MCFG, Micafungin; FCZ, Fluconazole; OSV, Oseltamivir; CAZ, Ceftazidime; CSFG, Caspofungin.

**Figure 1:**
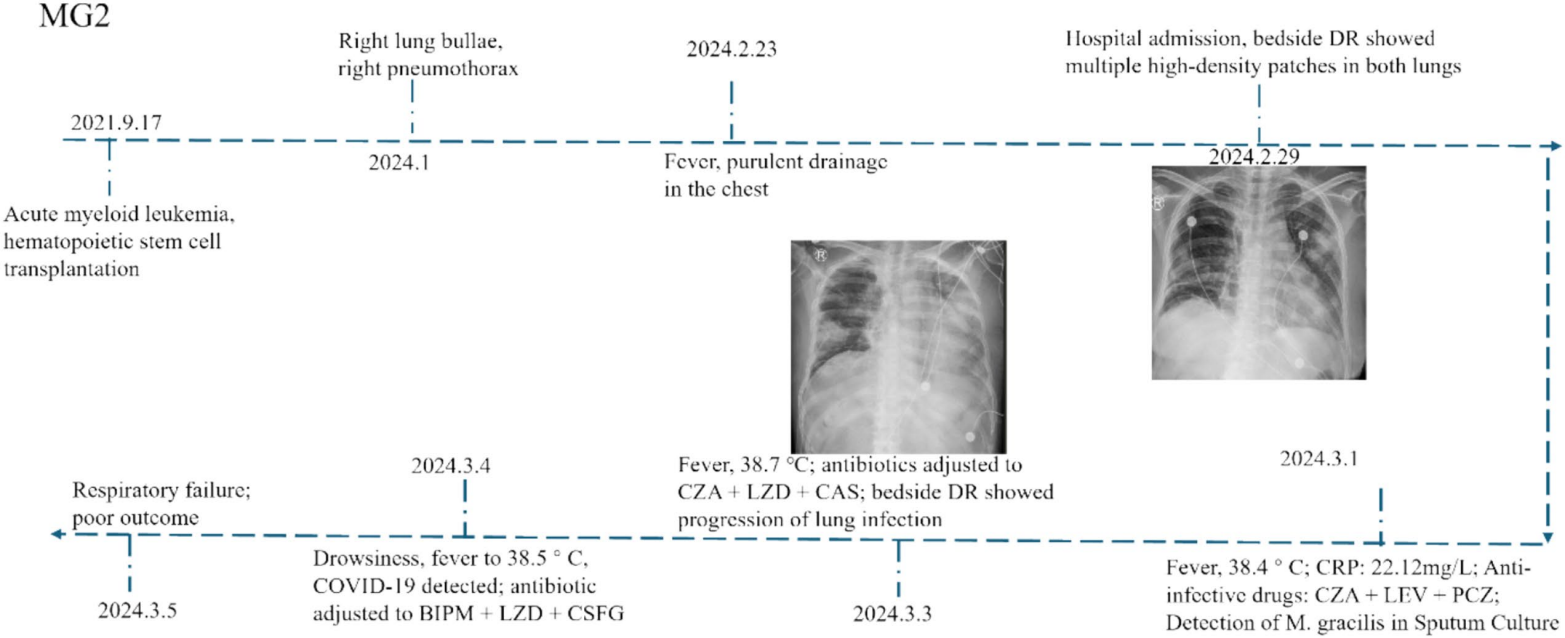
Disease progression timeline of pulmonary infection with *Microascus* spp. in patient MG2.

Compared with other cases, MG2 showed both typical disease characteristics and unique clinical features, we selected one representative case (MG2) for detailed analysis. The patient was initially diagnosed with *M. gracilis* infection during hospitalization, followed by concurrent 2019-nCoV infection. The patient’s post-transplantation status combined with mixed infection likely caused the poor clinical outcome. The disease progression then unfolded as follows. Patient MG2 who had undergone allogeneic HSCT 2 years prior developed pulmonary complications in January 2024, manifesting as right-side pneumatoceles with concomitant pleural effusion and radiologically confirmed pulmonary infection. On February 23, 2024, the patient presented with recurrent fever and purulent transformation of pleural effusion. Moreover, chest X-ray demonstrated multiple patchy consolidations with air bronchograms in bilateral lung fields (Figure 1). The patient developed persistent fever during hospitalization. Microbiological analysis of sputum cultures identified *M. gracilis*, with concurrent COVID-19 coinfection. Despite aggressive treatment with broad-spectrum antibiotics (bipenem, linezolid, micafungin) and molnupiravir, the patient’s condition deteriorated. The patient subsequently developed acute respiratory distress syndrome (ARDS) requiring mechanical ventilation.

### Comparison of patients infected with *Microascus* spp. in the literature and our study

Between 1992 and 2023, 16 cases of *Microascus* infection were reported in studies indexed in PubMed, with a median age of 56 years and no clear sex predominance. Among these cases, immunocompromised patients accounted for the majority (9/16), and disseminated infections-particularly in this group-were associated with high mortality (5/9), as shown in Table 3. *Microascus* often demonstrates reduced *in vitro* susceptibility to common antifungals, underscoring the need for susceptibility-guided therapy. Comparison with our cohort of 10 patients reinforces that pulmonary infections occur predominantly in severely immunocompromised individuals, such as patients with hematologic malignancies or lung transplant recipients, and often present as mixed infections. Breakthrough infections despite posaconazole prophylaxis highlight the importance of comprehensive microbiologic testing-including BALF metagenomic next-generation sequencing (mNGS) and culture - and tailored antifungal coverage.

**Table 3:** Summary of patients with *Microascus* spp. in the literature.

Case number	First author/year/country	Age/sex	Pathogen	Infection site	Underlying condition/transplant type	Treatment	Outcome	Ref.
1	De Vroey C/1992/Belgium	56/F	*M. cirrosus*	Onychomycosis (toenails)	Immunocompetent	IMD; GRF; KCZ	Treatment failed	de Vroey et al. (1992)
2		63/F	*M. cirrosus*	Onychomycosis (toenails)	Immunocompetent (arthrosis)	IMD; GRF; KCZ	Treatment failed	
3	Baddley JW/2000/United States	21/F	*M. cinereus*	Brain abscess	Allogeneic bone marrow transplant for aplastic anemia	Surgery; AMB, ITC	Deteriorated	Baddley et al. (2000)
4	Ustun C/2006/United States	49/M	*M. cirrosus*	Pneumonia	AML	Surgery; VCZ, AMB, Terb, G-CSF	Improved	Ustun et al. (2006)
5	Miossec C/2011/France	36/M	*M. cirrosus*	Disseminated (pleural fluid, intrapericardial fluid, blood clots, bronchial secretions, fungemia)	Heart and bilateral lung transplant for cystic fibrosis	VCZ, CSFG	Deteriorated	Miossec et al. (2011)
6	Schoeppler KE/2015/United States	64/M	*M. trigonosporus*	Pulmonary infection	Bilateral lung transplant for idiopathic pulmonary fibrosis	PCZ, AMB	Deteriorated	Schoeppler et al. (2015)
7	Taton/2017/Belgium	60/F	*M. cirrosus*	Tracheobronchial tree (left upper lobe bronchus, right intermediate bronchus)	Bilateral lung transplant for severe emphysema	Surgery; VCZ, CSFG, Terb, AMB; Reduction of immunosuppression	Cure	Taton et al. (2017)
8	Gao L/2018/China	17/F	*M. cirrosus*	Primary cutaneous infection (left ankle)	Immunocompetent (history of corticosteroid use)	ITC	Improved	Gao et al. (2018)
9	Ding Y/2020/United States	65/M	*M. gracilis*	Disseminated (pleura, lung, heart, brain, potential eyes)	Bilateral lung transplant for idiopathic pulmonary fibrosis	VCZ, AMB, MCFG, ISA, Terb	Deteriorated	Ding et al. (2020)
10	Malik/2020/India	13/M	*M. cinereus*	Brain (right frontoparietal abscess)	Immunocompetent child; No underlying risk factors	Surgery; AMB, followed by VCZ	Cure	Malik et al. (2020)
11	Los-Arcos I/2021/Spain	59/M	*M. senegalensis*	Tracheobronchitis	Bilateral lung transplant for COPD	MCFG, Terb, bronchial stent, bronchial dilations	Improved	Los-Arcos et al. (2021)
12	Liu Q/2021/China	72/F	*M. cirrosus*	Pulmonary infection	Bronchiectasis (immunocompetent)	VCZ, AMB	Improved	Liu et al. (2021)
13	Mhmoud NA/2021/Sudan	50/F	*M. gracilis*	Mycetoma (left foot)	Immunocompetent	Surgery; ITC	Improved	Mhmoud et al. (2021)
14	Faure E/2024/France	17/M	*M. melanosporus*	Bronchopulmonary infection	Multiple trauma (immunocompetent)	Olorofim, Terb	Improved	Faure et al. (2023)
15		61/M	*M. cirrosus*	Bronchopulmonary infection	Lung transplant	Olorofim	Improved	
16		65/M	*M. cirrosus*	Bronchopulmonary infection	Lung transplant	Olorofim, Terb	Deteriorated	

AML, Acute myeloid leukemia; IMD, Imidazole; GRF, Griseofulvin; KCZ, Ketoconazole; AMB, Amphotericin B; ITC, Itraconazole; ISA, Isavuconazole; VCZ, Voriconazole; PCZ, Posaconazole; MCFG, Micafungin; CSFG, Caspofungin; Terb, Terbinafine.

### Morphological characterization

**Figure 2:**
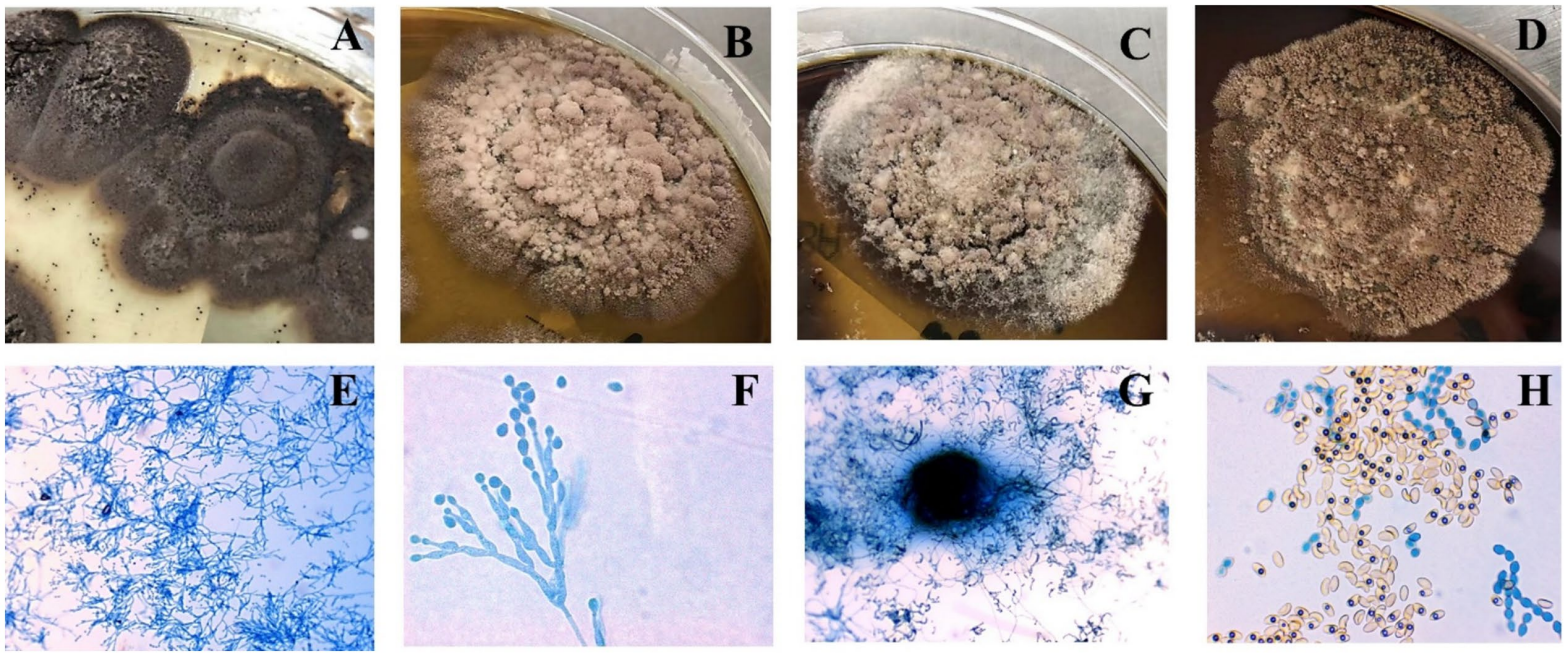
Colonial morphology **(A–D)** and lactophenol cotton blue-stained microscopic features **(E–H)** of *Microascus* spp. (**E,G** x400; **F,H** x1000).

Seven *M. gracilis* and one *M. cirrosus* isolate formed concentric olive-black colonies with floccose hyphae and dark granules after 14 days of SDA incubation (Figures 2A–D). Lactophenol cotton blue staining revealed septate hyphae, basally swollen conidiophores, and chain-forming oval/pear-shaped conidia (Figures 2E,F). Cleistothecia containing smooth reniform ascospores were observed in *M. cirrosus* (Figures 2G,H) (Cheng et al., 2023).

### Antifungal susceptibility patterns

All isolates exhibited elevated MIC to amphotericin B, fluconazole, and flucytosine (MIC >64 μg/mL) but were highly susceptible to terbinafine (MIC ≤0.125 μg/mL) (Table 4). Caspofungin demonstrated strain-dependent activity (MIC = 0.5–16 μg/mL). Among the nine patients who underwent HSCT, antifungal prophylaxis/therapy regimens included posaconazole (*n* = 6), voriconazole (*n* = 2), and isavuconazole (*n* = 1). Following *Microascus* infection diagnosis, all patients maintained their initial triazole antifungal therapy. Of these, two received adjunctive micafungin, four received caspofungin (of whom three exhibited disease progression and clinical deterioration).

**Table 4:** *In vitro* antifungal susceptibility profiles of eight *Microascus* isolates (MIC, μg/mL).

Antifungal agent	MG1	MG2	MG3	MG4	MG5	MG6	MG7	MG8
Caspofungin	0.5	8	8	8	8	2	8	>16
Terbinafine	0.125	0.125	0.125	0.125	0.125	0.125	0.125	0.125
Amphotericin B	>64	>64	>64	>64	>64	>64	>64	>64
Fluconazole	>64	>64	>64	>64	>64	>64	>64	>64
Flucytosine	>64	>64	>64	>64	>64	>64	>64	>64

### MALDI-TOF profiling and strain typing

**Figure 3:**
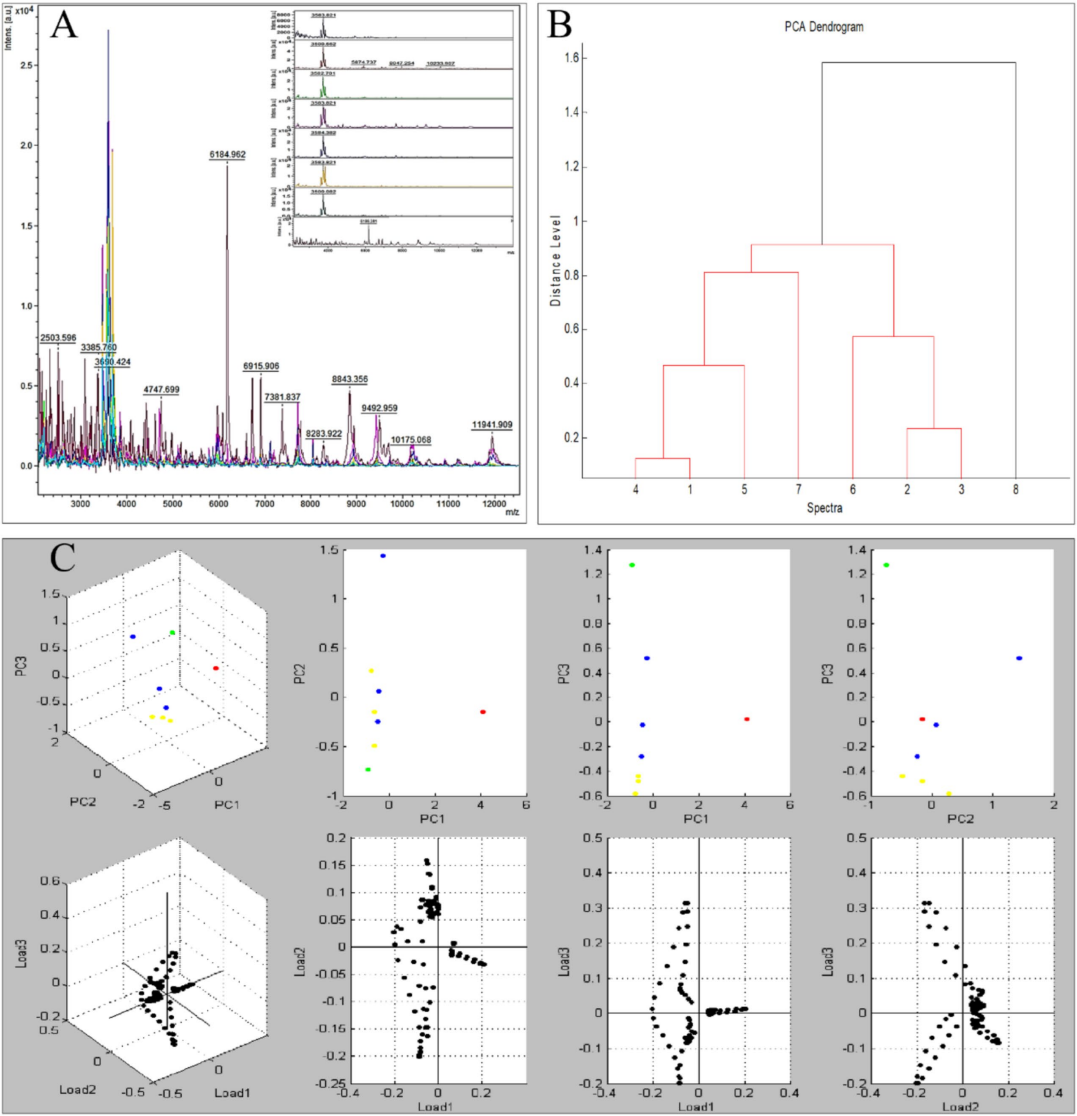
Protein fingerprinting and principal component analysis of eight *Microascus* strains. (**A**: protein fingerprint patterns of eight *Microascus* strains; **B**: cluster analysis of eight *Microascus* strains; **C**: principal component analysis of eight *Microascus* strains).

Protein fingerprinting distinguished two clusters: MG1-MG7 shared conserved peaks (3–4 high-intensity signals at 3400–3600 m/z; 1–2 low-intensity peaks at 2000–2200 m/z), while MG8 exhibited a unique 6,186 m/z signature (Figure 3A). Principal component analysis (PCA) and hierarchical clustering confirmed the taxonomic divergence between these clusters (Figures 3B,C).

### Phylogenetic relationships and species delineation

**Figure 4:**
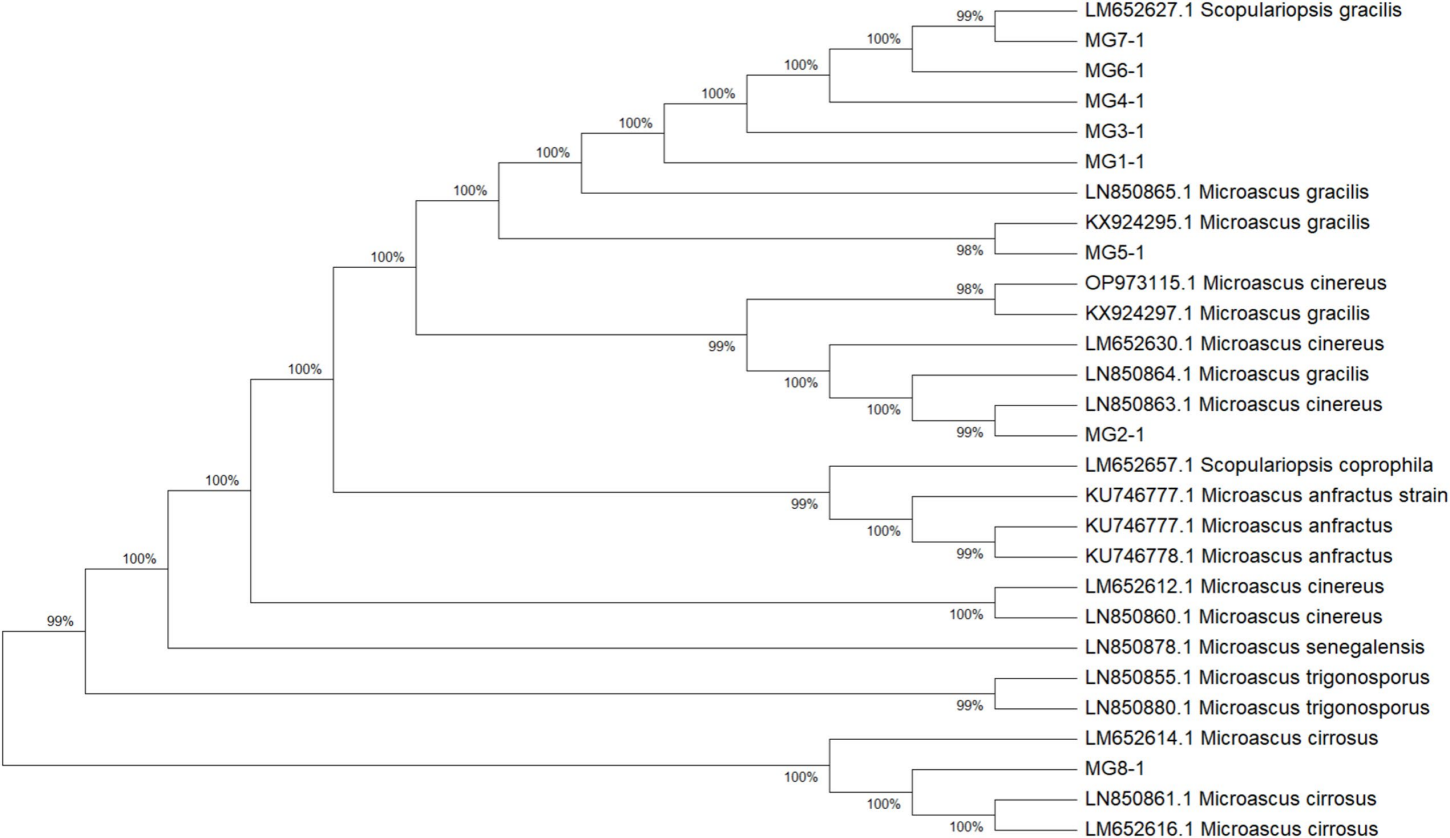
Phylogenetic analysis of the 8 *Microascus* isolates. (MG1-MG8: *Microascus* strains isolated in the present study; other strains: reference sequences obtained from GenBank http://www.ncbi.nlm.nih.gov/genbank/).

The multilocus phylogenetic analysis based on ITS, EF-1α, and TUB loci revealed remarkable interspecific diversity within *Microascus*. Notably, a highly supported monophyletic clade comprising reference strains of *M. gracilis* and clinical strains MG1/MG3–7 validated their taxonomic classification (Figure 4). Strain MG2 occupied a distinct phylogenetic position between the *M. gracilis* and *M. cinereus* clades, necessitating whole-genome sequencing for definitive classification. Furthermore, strain MG8 showed close phylogenetic affinity with the *M. cirrosus* reference strain despite minor branch length variations, which did not affect its taxonomic assignment.

### Environmental sampling of air and surfaces tested negative for *Microascus* spp

**Figure 5:**
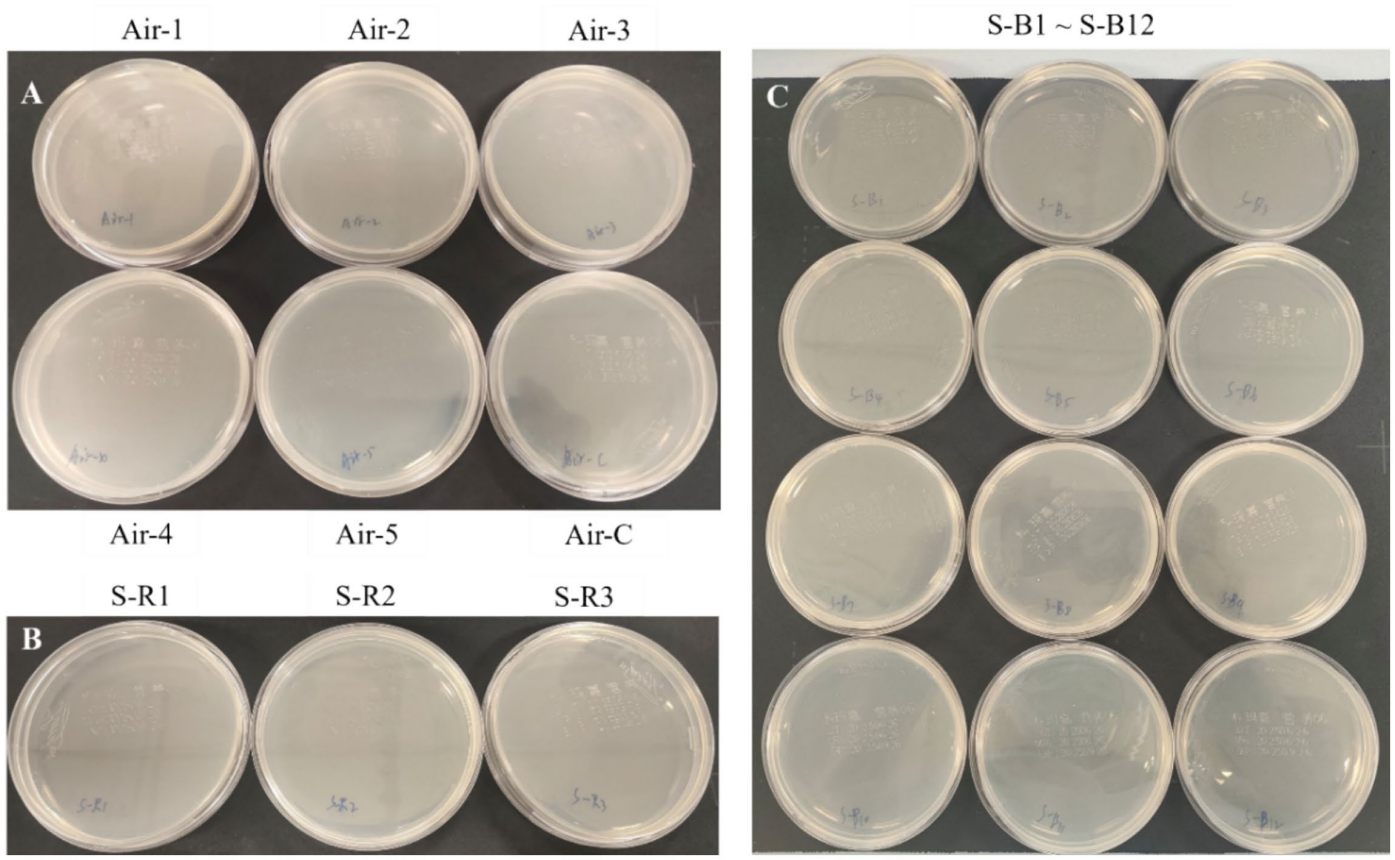
Targeted environmental sampling of air and surfaces in bronchoscopy suites and patient wards. (**A**: Settling bacterial monitoring of the air in the bronchoscopy operating room; **B**: surface monitoring of the bronchoscopy operating table; **C**: surface monitoring of the patient wards).

We performed targeted environmental sampling of air and surfaces in bronchoscopy suites and patient wards. Culture results showed no detectable *Microascus* spp. in the following samples: bronchoscopy suite air samples (Figure 5A), surfaces of bronchoscopy procedure tables (Figure 5B), and high-touch surfaces in patient wards (Figure 5C).

## Discussion

The occurrence of severe pneumonia during the stable phase of hematopoietic stem cell transplantation (HSCT) represents a clinically significant challenge. Current evidence implicates multiple risk factors, including environmental distribution of pathogens, host immunocompromised status, and prior antifungal exposure. In our cohort, *Microascus* ranked third most prevalent fungal genus in HSCT recipients, following *Aspergillus* and *Penicillium* spp. While existing literature predominantly documents *Microascus* infections as sporadic cases or reviews, this pathogen demonstrates concerning clinical versatility, capable of causing cutaneous infections or invasive dissemination to critical organs (heart, lungs, kidneys, and brain) with fatal outcomes (Miossec et al., 2011; Patterson et al., 2016; Eisman and Sinclair, 2014; Skiada et al., 2018; Baddley et al., 2010). A comparative analysis of 26 patients-16 from the literature and 10 from our study-regarding clinical features (age, sex, underlying diseases, infection site), treatment strategies, and outcomes revealed that *Microascus* infections can occur in immunocompetent individuals, typically presenting as localized infections of the skin, nails, or foot. However, these infections are more prevalent in immunocompromised patients, particularly those with solid organ transplants, mainly lung, or hematopoietic stem cell transplant recipients, and are frequently associated with pulmonary or disseminated infections that lead to high mortality. Both infection site and baseline immune status were identified as critical prognostic factors. Early intervention, including prompt diagnosis and treatment initiation, and individualized therapy are essential. Localized infections may be managed with surgical treatment combined with antifungal therapy, whereas disseminated or mixed fungal infections require aggressive combination antifungal regimens and, where applicable, reduction of immunosuppressive therapy.

Of particular relevance to our cohort, HSCT recipients were maintained on long-term cyclosporine-based immunosuppression for graft-versus-host disease prophylaxis alongside routine posaconazole/voriconazole prophylaxis. This therapeutic landscape likely selects for resistant *Microascus* strains, potentially explaining the observed fulminant fungal pneumonias. Radiological findings consistently revealed characteristic infectious patterns, including bronchovascular bundle thickening and cavitary lesions. Meanwhile, we evaluated the impact of the timing of antifungal therapy initiation on patient outcomes. Among the 10 patients studied, 9 were HSCT recipients, of whom 7 received prolonged prophylactic antifungal therapy initiated pre-admission or at admission, resulting in favorable outcomes. Conversely, the three cases with clinical deterioration comprised two HSCT patients who began antifungal treatment within 24 h post-admission and one non-HSCT patient with delayed initiation at 7 days post-admission. This suggests that early or prophylactic initiation correlates with improved outcomes, aligning with causal inference principles regarding exposure and outcome relationships, although this association does not confirm causation.

Notably, *Microascus* was detected within 10 days of admission in all cases, frequently alongside cytomegalovirus, *Pseudomonas aeruginosa*, or *Aspergillus* spp. The three fatal cases demonstrated synergistic morbidity with influenza A virus, 2019-nCoV, or *Nocardia* co-infections. The poor outcomes observed were primarily influenced by profound immunosuppression due to HSCT-related immune reconstitution failure and chronic comorbidities impairing antifungal efficacy in all three cases; additionally, one patient experienced COPD exacerbations compromising pulmonary defenses, and viral coinfections such as COVID-19 or influenza A triggered cytokine storms and multi-organ failure, further amplifying pathophysiological deterioration. This aligns with evidence that viral pathogens in immunocompromised hosts exacerbate immune dysfunction, creating a permissive environment for polymicrobial infections and poor outcomes (Vanderbeke et al., 2018; Arastehfar et al., 2020; Uhde et al., 2010).

Despite standard posaconazole/voriconazole prophylaxis, breakthrough *Microascus* infections occurred in our hematopoietic stem cell transplantation (HSCT) cohort, suggesting that conventional azole-based prophylactic regimens may be inadequate against this pathogen. All eight isolates exhibited elevated MICs (>64 μg/mL) to azoles, polyenes, and flucytosine, indicating potential intrinsic resistance mechanisms. In contrast, terbinafine demonstrated excellent *in vitro* activity (MIC ≤0.125 μg/mL across all strains). Heterogeneous susceptibility to echinocandins was observed, as reflected by variable MIC values to caspofungin among the isolates. These findings are consistent with those reported by Skóra et al. (2015), who also documented strong resistance of *Microascus* to single antifungal agents such as terbinafine, caspofungin, and posaconazole, with generally high MICs. Notably, their study revealed significant synergistic effects with triple combination therapy (terbinafine, caspofungin, and posaconazole) in vitro. Our clinical observations further support these results: a dose-escalated voriconazole regimen combined with caspofungin showed efficacy in partial responders, underscoring the potential of combination therapy to overcome monotherapeutic resistance. However, suboptimal responses were still observed in some cases even with susceptible agents, which may be attributed to concurrent viral or *Aspergillus* co-infections. This complexity underscores the need for comprehensive therapeutic strategies that integrate host immune status, co-infecting pathogens, and antifungal resistance profiles. Further genomic studies are warranted to elucidate species-specific resistance determinants in *Microascus*.

Current fungal infection diagnostics still rely heavily on morphological identification. These methods are time-consuming and often inaccurate, which may result in missed or incorrect pathogen detection. However, the slow growth kinetics of *Microascus* and technical challenges in rapidly obtaining observable diagnostic structures significantly impede timely detection. Notably, six next-generation sequencing (NGS) analyses in our cohort failed to detect *Microascus* species, highlighting the technical limitations of metagenomics for rare fungi detection, especially when pathogen-specific genomic signatures are underrepresented in reference databases. These diagnostic difficulties enhance the clinical significance of our findings, as better characterization of *Microascus* epidemiology and resistance patterns directly guides therapeutic decision-making for HSCT recipients during the stabilization phase. Our data underscore the necessity for developing rapid molecular diagnostics targeting conserved genomic regions.

The *Microascus* isolates in our study exhibited grayish-brown to black pigmentation with conidial morphological features consistent with established descriptions of the *Microascus* morphology (Liu et al., 2021). The observed morphological plasticity (melanized conidia and cleistothecial structures) suggests evolutionary adaptations related to environmental persistence or pathogenic regulation, though the underlying mechanisms remain unclear (Skiada et al., 2018). MALDI-TOF mass spectrometry proved highly effective for fungal identification, showing 100% concordance (7/7 strains) in genus-level classification based on characteristic protein fingerprints (3400–3,600 m/z spectral peaks) (Patel, 2015; Wei et al., 2022). However, species-level discrimination required supplementary phylogenetic analysis using multilocus sequence data. This highlights the necessity of combined approaches, whose diagnostic complexity warrants careful consideration. Notably, the phenotypic similarities among *Microascus*, *Aspergillus*, and *Penicillium* species necessitate implementing a tiered diagnostic algorithm in clinical laboratories. We propose a hierarchical identification framework incorporating: Morphological screening + MALDI-TOF + Multilocus sequence typing (MLST).

Our data suggest *Microascus* spp. as emerging opportunistic pathogens in post-hematopoietic stem cell transplantation (HSCT) invasive fungal infections, demonstrating a secondary prevalence rate to *Aspergillus* and *Penicillium* spp. This epidemiological pattern warrants heightened clinical vigilance, particularly because therapeutic complexities emerge from both frequent polymicrobial coinfections and intrinsic antifungal resistance. The technical challenges in species identification necessitate a multidimensional diagnostic protocol: (1) Prolonged incubation (5–14 days) for cleistothecia development; (2) MALDI-TOF MS spectral analysis focusing on the characteristic range; (3) Multilocus sequence typing (MLST) targeting *β-tubulin*, *ITS*, and *CAL* loci for definitive speciation. Antifungal susceptibility profiles revealed universal resistance to triazoles and echinocandins, while terbinafine showed the lowest MIC values (≤0.125 μg/mL). These data underscore the urgency to establish species-specific interpretive criteria for *Microascus*, distinct from existing guidelines for *Aspergillus* or *Candida*.

To investigate potential nosocomial sources of *Microascus* spp., targeted environmental sampling of air and surfaces in bronchoscopy suites and patient wards was performed. Although these fungi are ubiquitously distributed in natural environments—including soil, decaying plant materials, animal feces, and moist habitats—all environmental specimens tested negative for *Microascus* spp. by culture. This finding suggests an absence of detectable contamination within the examined healthcare settings during the study period. However, temporal and spatial variations in fungal load may occur, and the inherent limitations of conventional culturing in detecting low-abundance spores must be considered. These negative culture results, while not fully defining transmission dynamics, support excluding hospital-based environmental exposure as a source of these infections. If consecutive cases of *Microascus* spp. detection occur in the same clinical unit, surveillance involving multiple sampling locations and time points in high-risk areas, combined with molecular genotyping techniques for source attribution, should be prioritized.

The novel contributions of this study include the development of a tiered diagnostic strategy integrating morphological, MALDI-TOF MS, and MLST for accurate identification of *Microascus*; evidence-based recommendations for prophylaxis adjustment informed by *in vitro* susceptibility and literature review; and environmental surveillance data supporting targeted infection control. These findings provide critical insights for early detection and tailored management of *Microascus* infections in high-risk populations. Nevertheless, our study has three main limitations. First, its single-center design and small sample size (10 patients) limit the generalizability of the findings, particularly to non-HSCT populations. Second, the lack of molecular resistance data (e.g., *ERG11* mutations, efflux pump expression) constrains the mechanistic interpretation of antifungal resistance. Third, the absence of fungal burden quantification and autopsy data impedes accurate correlation of pathogen load with clinical outcomes and precise attribution of mortality. To address these issues, future studies should incorporate molecular profiling (e.g., whole-genome sequencing), standardize specimen processing, and implement fungal quantification methods (e.g., qPCR). Furthermore, enhanced autopsy efforts with ethical compliance are needed to clarify mortality causes. Prioritized research directions include developing rapid molecular diagnostics for high-risk groups, establishing multicenter surveillance to monitor resistance, and exploring combination therapies that leverage unique antifungal mechanisms such as those of terbinafine.

The increasing incidence of severe pulmonary *Microascus* infections, particularly among immunocompromised patients, is accompanied by high treatment failure rates and significant mortality. This is further compounded by the pathogen’s pan-resistance to conventional antifungal agents, underscoring an urgent need for improved early detection, elucidation of resistance mechanisms, and development of novel therapeutic strategies. In response, our future perspectives emphasize several key directions: First, diagnostic accuracy must be enhanced through the integration of *Microascus*-specific protein fingerprints into MALDI-TOF-MS databases and standardization of mNGS reporting protocols to facilitate early and reliable identification. Second, due to the high *in vitro* resistance observed, although resistance patterns are not fully defined, empirical antifungal susceptibility testing remains critical for tailoring targeted therapy. This is especially important for high-risk groups, such as transplant recipients receiving prophylaxis with azoles (e.g., posaconazole, voriconazole). Clinical vigilance should be heightened in these populations, incorporating multimodal data to differentiate colonization from invasive infection. Finally, strengthened environmental surveillance in high-risk hospital areas is essential to control potential nosocomial transmission. Collectively, these strategies aim to improve clinical outcomes through earlier intervention, individualized treatment, and evidence-based infection control in the face of this emerging and challenging pathogen.

## Data Availability

The datasets presented in this study can be found in online repositories. The names of the repository/repositories and accession number(s) can be found below: https://www.ncbi.nlm.nih.gov/genbank/, PRJNA835605.
